# Aspiration Thrombectomy Versus Stent Retriever Thrombectomy Alone for Acute Ischemic Stroke: A Systematic Review and Meta-Analysis

**DOI:** 10.7759/cureus.8380

**Published:** 2020-05-31

**Authors:** Marium Zafar, Muhammad Mussa, Roha S Memon, Shahrukh Nadeem, Muhammad S Usman, Javed Siddiqi, Alexander Norbash, Faisal Khosa, Vincent M Figueredo, Richard Krasuski, Muhammad S Khan

**Affiliations:** 1 Internal Medicine, Dow Medical College, Pakistan, Karachi, PAK; 2 Internal Medicine, Dow Medical College, Dow University of Health Sciences (DUHS), Karachi, PAK; 3 Internal Medicine, Dow University of Health Sciences (DUHS), Karachi, PAK; 4 Medicine, Dow University of Health Sciences (DUHS), Karachi, PAK; 5 Internal Medicine, Civil Hospital Karachi, Dow University of Health Sciences (DUHS), Karachi, PAK; 6 Neurosurgery, Desert Regional Medical Center, Palm Springs, USA; 7 Neurosurgery, Riverside University Health System Medical Center, Moreno Valley, USA; 8 Neurosurgery, Arrowhead Regional Medical Center, Colton, USA; 9 Neurosurgery, California University of Science and Medicine, Colton, USA; 10 Radiology, Neuroradiology, Interventional Radiology, University of California, San Diego, USA; 11 Radiology, Vancouver General Hospital, University of British Columbia, Vancouver, CAN; 12 Cardiovascular Diseases, Einstein Medical Center, Philadelphia, USA; 13 Cardiovascular Medicine, Duke University Health System, Durham, USA; 14 Internal Medicine, John H Stroger J. Hospital of Cook County, Chicago, USA

**Keywords:** aspiration thrombectomy, adapt, stent retriever thrombectomy, acute ischemic stroke

## Abstract

Introduction

This meta-analysis was conducted to assess the safety and efficacy of aspiration thrombectomy versus stent retriever thrombectomy for acute ischemic stroke (AIS).

Methods

We queried online databases for original studies comparing aspiration thrombectomy with stent retriever thrombectomy in patients with AIS. After article selection, data were extracted on multiple baseline characteristics and prespecified endpoints. Dichotomous data were presented as risk ratios (RRs) and corresponding 95% confidence intervals (CIs); continuous data as mean differences and 95% CIs. The data were pooled using a random-effects model. Subgroup analysis was conducted based on study type, site of occlusion, and age.

Results

We shortlisted nine relevant studies (n=1453 patients; n=690 receiving aspiration thrombectomy and n=763 receiving stent retriever thrombectomy). Meta-analysis demonstrated no significant difference between the two groups in the rates of successful recanalization (RR: 0.96 [0.87, 1.06]; p=0.42), excellent functional outcome (RR: 0.90 [0.80, 1.01]; p=0.06), or procedure time (weighted mean difference (WMD): -5.39 minutes [-11.81, 1.04]; p=0.10). However, when removing the study by Nishi et al., sensitivity analysis resulted in a significant reduction in procedure time with aspiration (WMD: -11.01 [-15.54, -6.49]; p<0.0001). No significant difference was observed in safety outcomes, including all-cause mortality (RR: 0.82 [0.57, 1.19]; p=0.30), intracranial hemorrhage (RR: 0.93 [0.55, 1.59]; p=0.80), symptomatic intracranial hemorrhage (RR: 0.72[0.42, 1.21]; p=0.57), or embolization to new territory (RR: 0.71 [0.42, 1.19]; p=0.19). Subgroup analysis revealed that aspiration thrombectomy led to significantly better outcomes in patients with a mean age ≤65 (RR: 1.15 [1.03, 1.29]; p=0.001), and stent retriever thrombectomy led to increased recanalization success in patients with a mean age >65 (RR: 0.89 [0.80, 1.00]; p=0.05).

Conclusions

Our updated meta-analysis reveals that both aspiration and stent retriever thrombectomy are comparably effective in the management of AIS. Shorter procedure times may potentially be attained with aspiration thrombectomy, and outcomes with each procedure may be age-dependent.

## Introduction

The American Heart Association (AHA) recommends mechanical thrombectomy for acute ischemic stroke (AIS) in patients presenting within six hours of symptom onset and considers it reasonable for selected patients presenting as long as 16-24 hours following stroke onset [1]. It also specifies that stent retrievers should remain the first choice for thrombectomy due to its exceptional functional outcomes, although instrumentation complications, such as dissections and vasospasm, have been reported. Another procedure known as ‘A Direct Aspiration First Pass Technique’ (ADAPT) has gained recognition as an alternative approach that can potentially improve outcomes over stent retriever thrombectomy alone [2-4].

ADAPT (henceforth referred to as ‘aspiration thrombectomy’) involves the suction-assisted removal of a thrombus by the largest catheter that can be reasonably placed within the target vessel and permits stent retriever salvage utilization in case of aspiration failure. Studies comparing aspiration with stent retriever thrombectomy have yielded conflicting findings [5-9]. This meta-analysis aims to resolve the inconsistencies between individual studies and provide a holistic, well-powered assessment of the safety and efficacy of aspiration thrombectomy in comparison to stent retriever thrombectomy. Additionally, we conducted a range of subgroup analyses to assess whether findings differed according to patient demographics.

## Materials and methods

This meta-analysis conforms to the Preferred Reporting Items for Systematic Reviews and Meta-Analyses (PRISMA) and Cochrane guidelines [10-11]. PubMed, Cochrane CENTRAL, and Scopus were searched in May 2019 without time or language restrictions. The string of keywords used for the literature search and the search strategy has been shown in Table 1.

**Table 1:** Search strategy

Database	Search Strategy	Results
Pubmed	(Acute[All Fields] AND ("ischemia"[MeSH Terms] OR "ischemia"[All Fields] OR "ischemic"[All Fields]) AND ("stroke"[MeSH Terms] OR "stroke"[All Fields])) OR AIS[All Fields] AND ("thrombectomy"[MeSH Terms] OR "thrombectomy"[All Fields] OR ("aspiration"[All Fields] AND "thrombectomy"[All Fields]) OR "aspiration thrombectomy"[All Fields]) OR ADAPT[All Fields] AND (("stents"[MeSH Terms] OR "stents"[All Fields] OR "stent"[All Fields]) AND retriever[All Fields]) OR solitaire[All Fields] OR Trevo[All Fields]	1022
Cochrane	Acute ischemic stroke OR AIS AND aspiration thrombectomy OR ADAPT AND stent retriever OR solitaire OR Trevo	242
Scopus	( ( TITLE-ABS-KEY ( acute AND ischemic AND stroke ) OR TITLE-ABS-KEY ( ais ) ) ) AND ( ( TITLE-ABS-KEY ( aspiration AND thrombectomy ) OR TITLE-ABS-KEY ( adapt ) ) ) AND ( ( TITLE-ABS-KEY ( stent AND retriever ) OR TITLE-ABS-KEY ( merci ) ) )	179

Studies were included if they: (a) were either randomized controlled trials (RCTs) or cohort studies; (b) directly compared the effects of aspiration thrombectomy with stent retriever thrombectomy alone; (c) included adult patients with AIS due to large-vessel occlusion. Two independent investigators (MM and MZ) conducted the review, and a third investigator (RSM) was consulted for discrepancies. Articles were first screened based on title and abstract and then full text.

From the studies obtained, the following efficacy outcomes were extracted: (a) successful recanalization (defined angiographically as a modified Thrombolysis in Cerebral Infarction (mTICI) score of 2b/3 on the completion angiogram obtained at the end of the index procedure); and (b) excellent functional clinical outcome (defined as a modified Rankin Scale (mRS) score of 0-1 or 0-2 after 90 days). Additionally, the following safety outcomes were extracted: (a) all-cause mortality; (b) intracranial hemorrhage (ICH); (c) symptomatic ICH (sICH); and (d) embolization to new territory (ENT). Quality assessment of included RCTs and observational studies was assessed using the Cochrane Collaboration Risk of Bias Tool (Table 2) and the Newcastle Ottawa scale (Table 3), respectively [12-13].

**Table 2:** Quality assessment of the RCTs RCT: randomized controlled trial

	Lapergue, 2017 [7]	Turk III, 2019 [8]
Random generation sequence	Low risk of bias	Low risk of bias
Allocation concealment	Low risk of bias	Low risk of bias
Blinding	Low risk of bias	Low risk of bias
Incomplete outcome data	Low risk of bias	Low risk of bias
Selective reporting	Low risk of bias	Low risk of bias
Other bias	Low risk of bias	Low risk of bias

**Table 3:** Quality assessment of the observational studies * shows the number of stars given to the study. A study can be awarded a maximum of one star for each numbered item within the selection and exposure categories. A maximum of two stars can be given for comparability.

	Lapergue, 2016 [5]	Mokin, 2017 [14]	Mokin, 2016 [15]	Stapleton, 2017 [9]	Gerber, 2017 [6]	Maegerlein, 2017 [16]	Nishi, 2018 [17]
Selection							
Exposed cohort	1	1	1	1	1	1	1
Non-exposed cohort	1	1	1	1	1	1	1
Ascertainment of Exposure	1	1	1	1	1	1	1
Outcome of interest	1	1	1	1	1	1	1
Comparability	2	2	2	2	2	2	2
Outcome							
Assessment of outcome	1	1	1	1	1	1	1
Length of follow-up	1	1	1	1	1	1	1
Adequacy of follow-up	1	1	1	1	1	1	1
Total score	9*	9*	9*	9*	9*	9*	9*

All statistical analyses were carried out on Review Manager V.5.3. Dichotomous data were pooled using a random-effects model and presented as risk ratios (RR) with 95% confidence intervals (CI). For continuous data, means and standard deviations were used. Mean differences and 95% CIs were calculated for each study and pooled to derive the weighted mean difference (WMD) and corresponding 95% CI. The chi-square test was used to analyze the differences between subgroups based on the stratification of studies according to study type (RCTs vs. observational studies), site of occlusion (anterior circulation vs. posterior circulation), and age (>65 years and ≤65 years). Sensitivity analysis was conducted for all outcomes by removing each study to check if any individual study had a disproportionate effect on the results. Visual inspection of the funnel plot was used to assess publication bias. The I^2^ statistic was used to evaluate heterogeneity, and a value >75% was considered significant heterogeneity [18]. p-value ≤0.05 was considered significant.

## Results

**Figure 1:**
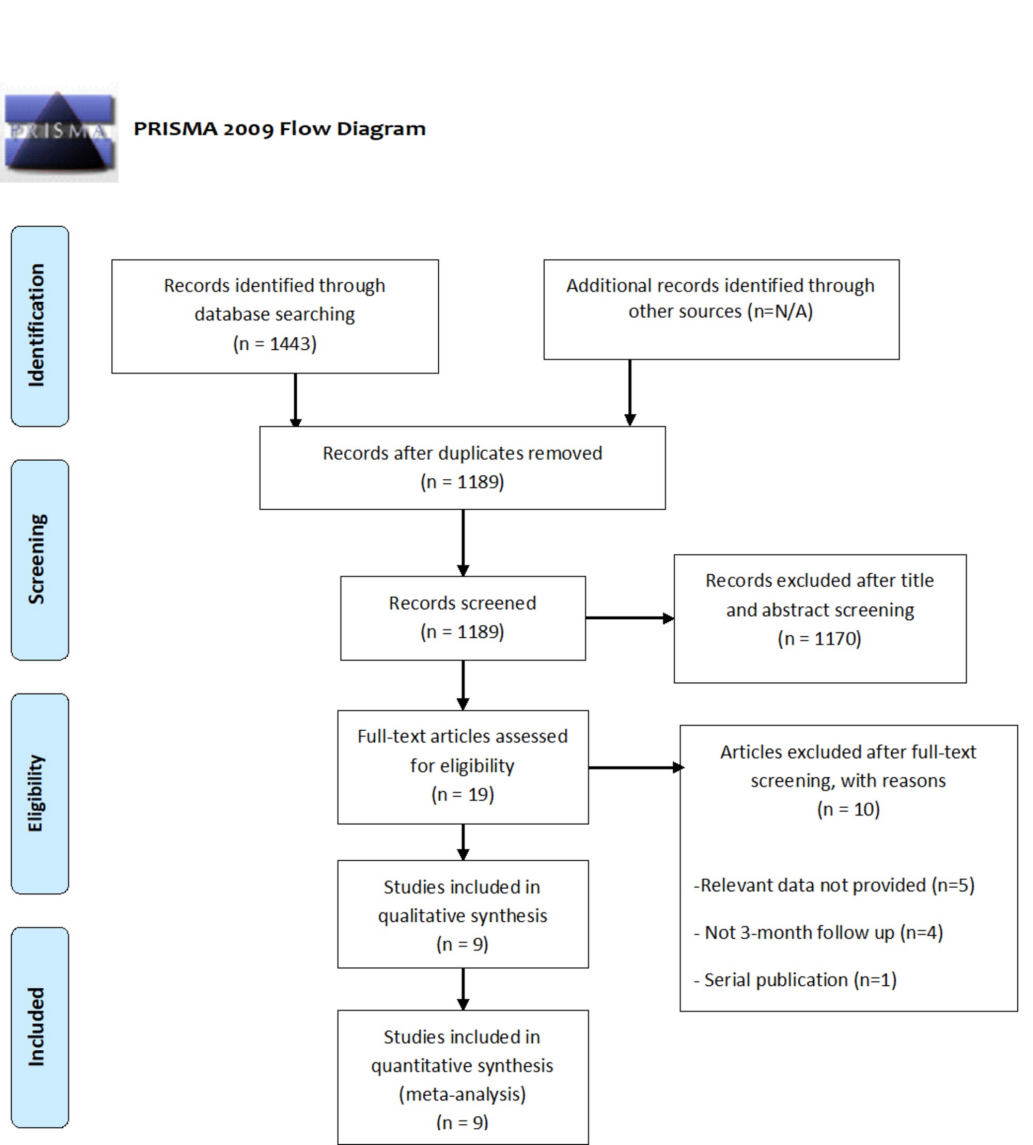
PRISMA flow chart.

Using the prespecified search strategy, our initial search retrieved 1443 studies. After exclusion, nine studies (two RCTs and seven observational studies) remained for the analysis. The PRISMA flow chart (Figure 1) outlines our literature search. These nine studies included 1453 AIS cases (n=690 in the aspiration thrombectomy arm and n=763 in the stent retriever thrombectomy arm). Baseline characteristics of studies and participants are presented in Table 4.

**Table 4:** Baseline characteristics of the studies included RCT = Randomized clinical trial, OS = Observational study, AC = Anterior circulation, ICA = Internal carotid artery, MCA = Middle cerebral artery, M1 = Middle cerebral artery branch M1, M2 = Middle cerebral artery branch M2, PC = Posterior circulation, BAO = Basilar artery occlusion, AP = Aspiration group, SR = Stent retriever group, NIHSS = National Institutes of Health Stroke Scale, mTICI = modified Thrombolysis in Cerebral Infarction, mRS = modified Rankin Scale, ICH = Intracranial hemorrhage, sICH = Symptomatic intracranial hemorrhage, ENT = Embolization to new territory

Study, Year	Study design	Study period	Location of occlusion	No of AP/(AP + SR)	Mean age, years	Men, no. (%)	NIHSS, mean	ASPECT, median	Inclusion criteria	Exclusion criteria	Efficacy outcomes	Safety outcomes
Lapergue et al, 2017 [7]	RCT	Oct 2015 - Oct 2016	AC (ICA, M1, M2)	192/381	69.9	207 (54.3)	16.2	7	1-Adults. 2-Imaging evidence of occlusion of the internal carotid. 3-M1 or M2 branches of the middle cerebral artery.	1-cerebral infarction of the posterior circulation. 2- occlusion of the cervical carotid artery. 3- prestrokemRS score >3.	1- Successful revascularization: mTICI score of 2b/3 on angiogram. 2- 90-day mRS score of 2 or less. 3- Change in NIHSS score at 24 hours.	1- Death at 90 days. 2- ICH at 24 hours. 3- sICH at 24 hours. 4- procedure-related adverse events.
Lapergue et al, 2016 [5]	OS	Nov 2012 - June 2014	AC (ICA, M1)	124/243	64.9	116 (47.7)	17	9	1- Proximal middle cerebral artery occlusion. 2- intracranial internal carotid artery occlusion. 3-Patients were eligible if they were treatable by MT within 6 hours of stroke onset, with bridging therapy (previous IV rtPA) or stand-alone thrombectomy.	1-Patients referred for acute ischemic stroke with cervical internal carotid occlusion/critical stenosis and basilar occlusion.	1- Successful recanalization defined angiographically as mTICI2b–3 on the angiogram at the end of the procedure. 2- mRS 0-2 at 90 days. 3- Early neurological improvement NIHSS score at 24 hours.	1- 90 days all cause mortality, 2- ENT. 3- sICH. 4- time: onset symptom to max mTICI score.
Mokin et al, 2017 [14]	OS	Mar 2012 - Mar 2016	AC (M2)	51/113	67	68 (58.1)	15	9	1- Treatment of patients within 24 h of stroke onset. 2- Isolated M2 occlusion.	1-Combined M1 and M2 occlusion and a tandem separate occlusion at a more proximal location (such as cervical ICA or ICA terminus occlusion). 2- strokes with an ischemic core of more than one-third of the MCA territory.	1- Successful recanalization: TICI score of 2b-3 and excellent recanalization was TICI 3. 2- 90 days mRS score of ≤2.	1- 3-months mortality. 2- parenchymal hematomas.
Mokin et al, 2016 [15]	OS	Mar 2012 - Jul 2015	PC	42/100	63.5	67 (67.0)	19.2	NA	1-Posterior circulation strokes.	1-Large brain stem strokes.	1- Successful recanalization: TICI score of 2b/3. 2- 90 days mRS score of ≤2.	1- ICH.
Stapleton et al, 2017 [9]	OS	Jun 2012 - Oct 2015	AC (NA)	47/117	67	61 (62.9)	16.5	8	1- Time from stroke onset or last seen well to groin puncture of less than 8 hours. 2- No acute hemorrhage. 3- occlusion of the terminal internal carotid artery (ICA) or middle cerebral artery (MCA) on CTA. 4- NIHSS score of 8 or higher. 5- Infarct volume <100 ml and/or ASPECTS > 4. 6- Baseline mRS score of 3 or less. 7- life expectancy greater than 6 months. 8-anatomy permitting catheterization of the intracranial vessels.	1- Patients with posterior circulation occlusions. 2- Patients with failed primary stentriever thrombectomy followed by successful aspiration thrombectomy.	1- Successful reperfusion was defined as a TICI score of 2b/3. 2- 90-day mRS score. 3- 7-day NIHSS score.	1- Death. 2- ICH. 3- sICH.
Gerber et al, 2017 [6]	OS	Jan 2013 - Apr 2016	PC	20/33	63	22 (67.0)	22	7	1- Presence of a BAO or an occlusion of the intracranial vertebral artery (ICVA, V4-segment) leading to a BAO. 2-EVT with SRT or AT.	1- Patients that required additional intracranial angioplasty or a permanent stent.	1- Successful recanalization: AOL 2 and 3.	1- Death. 2- ENT. 3- ICH. 4- hemorrhagic infarction. 5- parenchymatous hematoma.
Maegerlein et al, 2017 [16]	OS	Jun 2014 - Mar 2016	AC (ICA, MCA, ACA) + PC	36/97	74.5	52 (53.6)	NA	NA	1- Exclusive usage of either the ADAPT or the stent retriever technique. 2- Patients that had been treated by mechanical thrombectomy due to distal internal carotid artery (ICA)/carotid-T, middle cerebral artery (MCA), anterior cerebral artery (ACA), basilar artery (BA) occlusion.	1-Tandem occlusions 2-More than 1 clot. 3-Both procedures used. 4-Intracranial stenosis.	1- TICI scores after opening POS and after rescue maneuvers. 2- mRS scores at 90 days and at discharge.	1- Death. 2- ENT. 3- sICH. 4- Subarachnoid hemorrhage.
Nishi et al, 2018 [17]	OS	Sep 2014 - Mar 2015	AC + PC	44/89	75	60 (60.6)	18	NA	1-Tandem occlusions	1-Anterior circulation stroke and ASPECTS less than 5 and hemorrhagic infarction. 2-NIHSS score less than 4. 3-Time from symptom onset was more than 8 hours were excluded, except for those with wake-up stroke.	1- 90 days mRS score 0-2. 2- mTICI score 2b/3.	1- sICH
Turk III et al, 2019 [8]	RCT	Jun 2015 - Jul 2017	AC (ICA, MCA M1)	134/270	71.4	125 (46.3)	16.9	8	1- Age 18 and older. 2- NIHSS ≥8 at the time of neuroimaging 3- Patient Presenting within 6 hours of AIS from AC large-vessel occlusion. 4- Neuroimaging demonstrates large vessel proximal occlusion (distal ICA through MCA bifurcation) 5- Pre-event Modified Rankin Scale score 0-1.	1- Presence of an existing or pre-existing large territory infarction. 2- Known or suspected pre-existing (chronic) large vessel occlusion in the symptomatic territory. 3- Absent femoral pulses. 4- Excessive vascular access tortuosity. 5- Pregnancy. 6- Severe contrast allergy or absolute contraindication to iodinated contrast. 7-Chronic intracranial occlusion. 8- The patient has severe or fatal comorbidities.	1- 90 days mRS score 0-2. 2- mTICI score 2b/3. 3- changes in NIHSS score (24 hours, 1 week).	1- 90 days all cause mortality. 2- sICH. 3- ICH. 4- ENT and other clinically significant complications.

**Figure 2:**
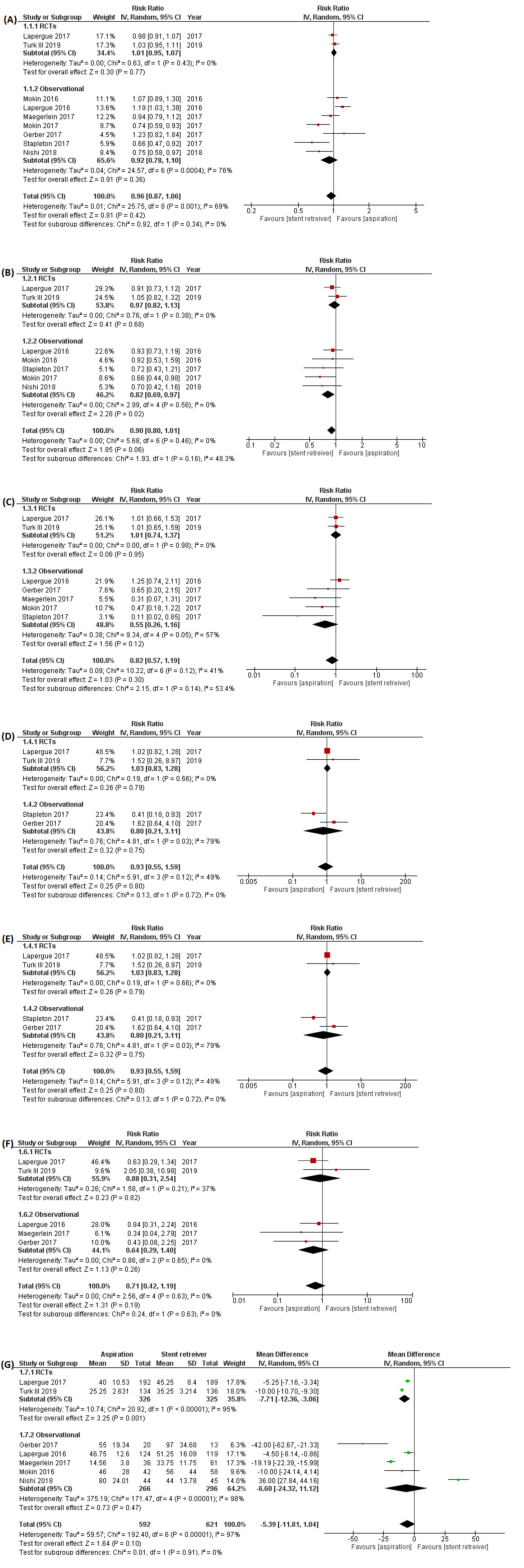
Forest plots for safety and efficacy outcomes (A) Successful recanalization (modified Thrombolysis in Cerebral Infarction; mTICI) score of 2b/3 on the completion angiogram obtained at the end of the index procedure; (B) Excellent functional outcome (modified Rankin Scale; mRS) score 0-1 or 0-2 after three months; (C) All-cause mortality; (D) Intracranial hemorrhage (ICH); (E) Symptomatic Intracranial hemorrhage (sICH); (F) embolization to new territory (ENT); (G) Procedure time (minutes). The diamond indicates the estimated relative risk [95% Confidence interval (CI)] for all patients. IV = Inverse variance

Our pooled analysis shows no significant difference between the aspiration group and the stent retriever group in efficacy outcomes: (a) successful recanalization (TICI 2b/3 at final angiogram) (RR: 0.96 [0.87, 1.06]; p=0.42; I2=69%); and (b) excellent functional outcome (RR: 0.90 [0.80, 1.01]; p=0.06; I2=0%). Similarly, there were no significant differences between the groups in the incidence of adverse outcomes, i.e.: (a) all-cause mortality (RR: 0.82[0.57, 1.19]; p=0.30; I2=42%); (b) ICH (RR: 0.93[0.55, 1.59]; p=0.80; I2=49%); (c) sICH (RR: 6 0.72[0.42, 1.21]; p=0.57; I2=0%), and (d) ENT (RR: 0.71[0.42, 1.19]; p=0.19; I2=0%). No significant difference in procedural time was appreciated between the two groups (WMD: -5.39 minutes [-11.81, 1.04]; p=0.10; I2=0%). However, results became significant by removing the study by Nishi et al. during the sensitivity analysis and favored the aspiration group (WMD: -11.01 [-15.54, -6.49]; p<0.0001; I2=44.3%) (Figure 2). Apart from this finding, the sensitivity analysis did not reveal any study that had a disproportionate effect on any outcome.

Subgroup analyses demonstrated no significant difference between any of the tested subgroups, with the following exception: aspiration thrombectomy led to significantly higher rates of successful recanalization in patients with a mean age ≤65 years (RR: 1.15 [1.03, 1.29]; p=0.001; I2=0%) while stent retriever thrombectomy led to increased recanalization success in patients >65 years of age (RR: 0.89 [0.80, 1.00]; p=0.05; I2=70%) (p-interaction=0.002). Table 5 and Table 6 show the results of the subgroup analysis of efficacy and safety outcomes, respectively.

**Table 5:** Results of efficacy outcomes stratified into subgroups according to type of study, site of occlusion, and age RCTs = Randomized clinical trials, AC = Anterior circulation; PC = Posterior circulation

	Successful recanalization			Functional outcome after 90 days		
	RR (95% CI)	p-value	I^2^	RR (95% CI)	p-value	I^2^
1. Type of study						
RCTs	1.01 (0.95, 1.07)	0.34	0	0.97 (0.82, 1.13)	0.16	48.3
Observational	0.92 (0.78, 1.10)			0.82 (0.69, 0.97)		
2. Site of occlusion						
AC	0.95 (0.84, 1.09)	0.20	39.3	0.90 (0.79, 1.03)	0.94	0
PC	1.10 (0.92, 1.31)			0.92 (0.53, 1.59)		
3. Age						
≤65 years	1.15 (1.03, 1.29)	0.002	90	0.93 (0.74, 1.16)	0.60	0
>65 years	0.89 (0.80, 1.00)			0.86 (0.72, 1.02)		

**Table 6:** Results of safety outcomes stratified into subgroups according to type of study, site of occlusion, and age AC = Anterior circulation; PC = Posterior circulation, ICH = Intracranial hemorrhage, sICH = Symptomatic intracranial hemorrhage, ENT = Embolization to new territory. P-value for subgroup differences.

	All-cause mortality			ICH			sICH			ENT		
	RR (95% CI)	p-value	I^2^	RR (95% CI)	p-value	I^2^	RR (95% CI)	p-value	I^2^	RR (95% CI)	p-value	I^2^
1. Type of study												
RCTs	1.01 (0.74, 1.37)	0.14	53.4	1.03 (0.83, 1.28)	0.72	0	1.00 (0.52, 1.94)	0.1	63.4	0.88 (0.31, 2.54)	0.63	0
Observational	0.55 (0.26, 1.16)			0.80 (0.21, 3.11)			0.40 (0.17, 0.95)			0.64 (0.29, 1.40)		
2. Site of occlusion												
AC	0.90 (0.61, 1.33)	0.61	0	0.80 (0.40, 1.60)	0.23	31	0.74 (0.39, 1.40)	NA	NA	0.79 (0.45, 1.39)	0.5	0
PC	0.65 (0.20, 2.15)			1.62 (0.64, 4.10)			Not estimable			0.43 (0.08, 2.25)		
3. Age												
≤65	1.12 (0.69, 1.82)	0.18	43.4	1.62 (0.64, 4.10)	0.23	31	0.41 (0.11, 1.55)	0.37	0	0.71 (0.30, 1.64)	0.98	0
>65	0.70 (0.42, 1.16)			0.80 (0.40, 1.60)			0.79 (0.45, 1.41)			0.72 (0.35, 1.45)		

## Discussion

In summary, this meta-analysis demonstrated the following: (a) No significant differences exist between aspiration and stent retriever thrombectomy in achieving complete recanalization and excellent functional outcomes, as well as preventing adverse events; and (b) neither treatment was better in decreasing procedure time. However, once a sensitivity analysis was undertaken following the elimination of the study by Nishi et al., the pooled time duration of the aspiration thrombectomy was reduced by 5.62 minutes in comparison to stent retriever thrombectomy and became statistically significant. Outside of this example, results appeared robust and reliable, and outcomes were not overly influenced by any particular study. They further remained consistent during subgroup stratification, with the exception of patient age. Interestingly, patients aged over 65 years of age achieved better rates of successful recanalization with stent retrievers, whereas younger patients (≤65 years) had higher rates of recanalization with aspiration thrombectomy. The study-level nature of our meta-analysis means that this finding is exploratory and not definitive. However, the wide statistical difference between the two groups (p-interaction=0.002) strongly suggests an age-related effect, and future analyses of trials should ideally explore this association in depth.

It has been hypothesized that aspiration thrombectomy can potentially lead to better outcomes. Unlike the aspiration technique, the stent retriever technique requires passage through the clot, with a risk for clot fragmentation and distal embolization [19]. Some comparative observational lean in favor of aspiration thrombectomy, others concur with our findings and report no difference between the two interventions [5-6,14-15]. The latter studies have also support from two RCTs. The Contact Aspiration vs Stent Retriever for Successful Revascularization (ASTER) trial examined whether aspiration thrombectomy would result in superior recanalization, whereas the latest trial Comparison of Direct Aspiration versus Stent Retriever as a First Approach (COMPASS) assessed for the noninferiority of functional outcomes with aspiration compared to stent retriever thrombectomy [7-8]. Despite their differing hypotheses, ASTER and COMPASS came to similar conclusions on the comparable efficacy of the two treatments.

In contrast to our findings, a meta-analysis published prior to the COMPASS Trial concluded significantly better functional outcomes with aspiration thrombectomy [8,20]. Due to the limited amount of data availability, the authors called for further multi-center RCTs to validate the conclusion. Another meta-analysis by Primiani and colleagues used a different statistical approach to compare these interventions [21]. They conducted a single-arm, non-comparative meta-analysis of each intervention and compared the two as subgroups. This study included only the preliminary results of the COMPASS trial. Our study employs a relatively more rigorous statistical approach, involving a head-to-head comparison of the two interventions and, therefore, minimizes heterogeneity between the treatment and control groups. Additionally, we excluded small, non-comparative observational studies likely to introduce bias. The results of our study, however, are congruent with the findings reported by Primiani et al [21].

Procedure time, like reperfusion status, appears to be a predictor of good functional outcomes [22]. Moreover, a longer duration of thrombectomy is an independent risk factor for sICH [23]. Multiple studies evaluating procedure duration report faster thrombectomy using aspiration than was observed with stent retrievers [9,16,24]. In contrast to most studies, the observational study by Nishi et al. concluded that aspiration thrombectomy resulted in longer procedural times [17]. This study appeared to have a significant influence on our meta-analysis; and its removal during the sensitivity analysis changed the results from non-significant to significantly in favor of aspiration. The difference can potentially be attributed to the relatively small sample size of this cohort or to differences in operator skill.

While the previous meta-analysis in this area reported a lower frequency of sICH in the aspiration thrombectomy group; in keeping with the recent COMPASS trial, our updated study found no significant differences in the incidences of adverse events in the two treatment groups [8,20]. We believe our analysis reinforces the significance of the COMPASS trial results [8].

Our study, limited by its exploratory nature, carries the inevitable bias associated with a study-level meta-analysis. Additionally, the results of this analysis were partially based on observational studies, which are relatively more susceptible to bias due to confounding.

## Conclusions

In this study, we compared the safety and efficacy of aspiration thrombectomy versus stent retriever thrombectomy among 1453 AIS patients. Additionally, we performed a subgroup analysis to evaluate whether the type of study, location of occlusion, and age of patient influenced the results produced. We also compared the procedure time of both the treatments. Our study concludes that both aspiration and stent retriever thrombectomy have comparable efficacy in the management of AIS, allowing the operator to individualize the treatment choice to the needs of the patient. These results are congruent with RCTs in the area and serve to consolidate and increase confidence in their findings. Procedure time may potentially be reduced with the use of aspiration thrombectomy, and outcomes with each procedure may be age-dependent.
